# Extracellular vesicles in the pathogenesis of rheumatoid arthritis and osteoarthritis

**DOI:** 10.1186/s13075-016-1178-8

**Published:** 2016-12-01

**Authors:** Joseph Withrow, Cameron Murphy, Yutao Liu, Monte Hunter, Sadanand Fulzele, Mark W. Hamrick

**Affiliations:** Department of Cellular Biology & Anatomy, Medical College of Georgia, Augusta University, Laney Walker Blvd. CB2915, Augusta, GA 30912 USA

**Keywords:** Extracellular vesicles, MicroRNA, Fibroblast-like synoviocyte, Chondrocyte, MMP-13, IL-1β, TNF-α

## Abstract

Osteoarthritis (OA) and rheumatoid arthritis (RA) are both debilitating diseases that cause significant morbidity in the US population. Extracellular vesicles (EVs), including exosomes and microvesicles, are now recognized to play important roles in cell-to-cell communication by transporting various proteins, microRNAs (miRNAs), and mRNAs. EV-derived proteins and miRNAs impact cell viability and cell differentiation, and are likely to play a prominent role in the pathophysiology of both OA and RA. Some of the processes by which these membrane-bound vesicles can alter joint tissue include extracellular matrix degradation, cell-to-cell communication, modulation of inflammation, angiogenesis, and antigen presentation. For example, EVs from IL-1β-stimulated fibroblast-like synoviocytes have been shown to induce osteoarthritic changes in chondrocytes. RA models have shown that EVs stimulated with inflammatory cytokines are capable of inducing apoptosis resistance in T cells, presenting antigen to T cells, and causing extracellular damage with matrix-degrading enzymes. EVs derived from rheumatoid models have also been shown to induce secretion of COX-2 and stimulate angiogenesis. Additionally, there is evidence that synovium-derived EVs may be promising biomarkers of disease in both OA and RA. The characterization of EVs in the joint space has also opened up the possibility for delivery of small molecules. This article reviews current knowledge on the role of EVs in both RA and OA, and their potential role as therapeutic targets for modulation of these debilitating diseases.

## Background

Osteoarthritis (OA) and rheumatoid arthritis (RA) are prevalent causes of morbidity and disability worldwide. OA is estimated to affect 3.8% (95% CI: 3.6–4.1) of the world’s population, with the United States having an even higher prevalence [1]. Characteristics of this disease include degraded cartilage, moderate synovial inflammation, alteration of bony structure, pain, and impaired mobility [2]. Pain management and weight loss provide some relief, yet these interventions do not halt the progression of the disease. Knee arthroplasty removes the arthritic tissue but 10–20% of patients still report that pain remains after surgery [3]. RA in northern Europe and the United States has a prevalence of 0.5–1% [4]. The disease is characterized by swelling, tenderness, and destruction of synovial joints [5, 6]. Auto-antibodies such as rheumatoid factor and anti-citrullinated protein antibody help to detect the presence of the disease before it presents clinically [7]. Disease-modifying anti-rheumatic drugs such as methotrexate and newer biologic agents have helped to improve the prognosis and prevent the progression of RA; however, despite the severity of both of these diseases, relatively little is known about the complex pathogenesis.

Recently, membrane-bound microparticles/microvesicles, apoptotic bodies, and exosomes—collectively known as extracellular vesicles (EVs)—have been identified and shown to carry microRNA (miRNA), mRNA, and protein [8–10]. The three broad categories of EVs are distinguished based on their biogenesis. Microparticles/microvesicles are formed by outward budding and fission of the plasma membrane [11]. Exosomes are created in the endosomal network of the cell and released by the fusion of multivesicular bodies with the plasma membrane [11]. Apoptotic bodies are formed as cells undergo apoptosis and release their contents in membrane-bound vesicles [11]. It is difficult to distinguish these three subgroups of EVs. Size was once thought to be a major determining factor, where vesicles larger than 100 nm were thought to be microvesicles and vesicles smaller than 100 nm were generally considered exosomes [12]. However, recent research has shown that the size of these particles overlaps with each other [12]. Protein composition determination is a major mechanism of identification of these particles and is the most widely accepted [13]. While protein composition is helpful, there is no single marker whose presence or absence can define the type of EVs [11]. Tetraspanins CD9, CD63, and CD81 were at one point thought to be specific to exosomes but this has now been disproven [11]. Establishment of multiple public databases that profile the protein composition of different EVs has helped with the identification process [14]. The term exosome is often used generally to reference these membrane-bound particles, and there has been a large increase in publications on exosomes since 2010 [13, 15]. In the absence of a standard approach to isolating exosomes sensu stricto, this review will refer to these small, membrane-bound particles as EVs.

EVs are known to function in cell-to-cell communication and are able to transmit their contents to different cells and cause various changes in cell transcription and cell proliferation [16–20]. They have also been shown to vary in their contents, specifically miRNA, in different disease states to the degree that a patient’s EV miRNA expression profile can serve as a potential biomarker [21–23]. Previous research indicates that EV content is altered in pathologic conditions of RA and OA [24, 25]. This review aims to highlight current knowledge on the role of EVs in OA and RA.

### Extracellular vesicles in the development and pathogenesis of RA

The role of EVs in the pathogenesis of RA is beginning to be better understood. Some of the pathogenic processes in which EVs have been implicated with regard to the development of RA include formation of immune complexes, antigen presentation, delivery of miRNA, inflammatory cytokines, proteases, and other proteins, activation of fibroblast-like synoviocytes (FLS), cell-to-cell communication, and degradation of the extracellular matrix (Table 1) [26].

**Table 1:** Proposed roles of extracellular vesicles in rheumatoid arthritis

Process	Description
Antigen presentation and immune complex formation	Present antigens for recognition by immune cells. Proteins such as DEK, vimentin, fibrin, fibronectin, fibrinogen, and AIM are present in the membrane. These become citrullinated and are thought to activate the innate and adaptive immune system, resulting in inflammation. Additionally these antibodies form to these complexes and deposit in the tissues, resulting in increased inflammation [27, 31, 35]
Inflammation	Carry membrane-bound TNF-α, which causes inflammation. EVs stimulate production of TNF-α, IL-6, IL-8, and mPGES-1, further increasing inflammation. Platelet-derived EVs are found in patients with RA and increase inflammation in an IL-1 receptor-mediated mechanism. Presence of EV-based immune complexes causes increased inflammation. EVs can activate TLR4, which triggers anti-inflammatory genes. EVs carry ANXA1 which reduces inflammatory cytokines [24, 36–38, 41, 43, 47]
Destruction of ECM	Carry catabolic proteases such as MMPs, ADAMTS-5, Hexosaminidase D, and B-glucuronidase. This causes the breakdown of ECM, resulting in the destruction of cartilage and more inflammation. ANXA1 in EVs activates anabolic genes in chondrocytes [47, 51–57]
Biomarker	Differences in content of synovial fluid and plasma EVs can serve as a biomarker for disease. There has proven to be an increased concentration of EVs in plasma of people with RA. Additionally, the presence of citrullinated proteins in EV membrane is a potential biomarker that is specific to RA [27, 41, 58]
Delivery of miRNA	Deliver miRNA to cells altering response to inflammation. Dendritic cells are known to secrete EVs with increased levels of miR-155 and miR-146a in response to inflammation [58–65]
Therapeutic	EVs derived from IL-10-treated dendritic cells have shown anti-inflammatory properties in patients with RA. EVs have also been created that can target the synovial membrane specifically. Demonstration that EVs have anti-inflammatory properties illustrates the possibility of mimicking that stimulation therapeutically [43, 47, 79–81]

*AIM* apoptosis inhibitor of the macrophage, *ANXA1* annexin A1, *DEK* DNA-binding protein, *ECM* extracellular matrix, *EV* extracellular vesicle, *miRNA* microRNA, *MMP* matrix metalloproteinase, *mPGES-1* microsomal prostaglandin E synthase 1, *RA* rheumatoid arthritis, *TLR4* Toll-like receptor 4

### Antigen presentation and immune complex formation

EVs present antigens that result in antibody formation characteristic of RA [27]. Detection of different autoantibodies offers different degrees of sensitivity and specificity for detecting RA. The presence of anti-cyclic citrullinated peptide (anti-CCP) antibodies in the serum has a specificity of 96% for the diagnosis of RA and is one of the most useful biomarkers currently available [7]. Citrulline is a neutral amino acid that is formed following deimination of protein-bound arginine by the peptidylarginine deiminase family of enzymes (PADs) [28]. PADs are a family of calcium-dependent enzymes that have been implicated in the pathogenesis of cancer progression and a wide range of autoimmune diseases [28]. FLS-derived EVs have been shown to carry citrullinated proteins such as fibrinogen components, vimentin, and apoptosis inhibitor of the macrophage (AIM) in their membrane [27]. This cargo stimulates antibodies to these proteins and the formation of immune complexes [27, 29–31]. Other studies have demonstrated that synovial fluid-derived EVs originating from different cells also form immune complexes. A large subset of the EV-based immune complexes found was CD41^+^, which is a platelet marker, suggesting that there is a large cohort of immunogenic EVs that are derived from platelets [31]. Immune complexes formed around EVs cause marked upregulation of leukotriene production from neutrophils in vitro, indicating that they contribute to inflammation [31]. Nielsen et al. also demonstrated EV-based immune complex formation in rheumatologic diseases. This study showed that plasma from patients with RA had significantly more IgM attached to circulating EVs than plasma from controls [32]. Deposition of immune complexes in tissues is recognized as one of the major mechanisms of inflammation in rheumatic diseases [31, 33, 34]. EVs have also been shown to function in antigen presentation [35]. EVs containing DNA-binding protein (DEK) are known to deliver this antigen to CD8^+^ lymphocytes and NK cells [35]. This results in a more efficient antigen presentation and can result in increased activation of the immune system [35].

### Inflammation

EVs derived from the joints of patients with RA have been shown to induce inflammatory changes in chondrocytes in vitro [24, 36]. A membrane-bound form of TNF-α is present in EVs derived from FLS isolated from RA patients [37]. Activation of FLS by the membrane-bound TNF-α in EVs results in activation of NF-κB which promotes inflammation and renders T cells found in the synovial tissue resistant to apoptosis [37]. Furthermore, EVs isolated from TNF-α-treated T cells and monocytes have been shown to stimulate FLS production of cyclooxygenase 2 (COX-2), microsomal prostaglandin E synthase 1 (mPGES-1), and prostaglandin E2 (PGE2) [38]. COX-2 converts arachadonic acid to inflammatory mediators such as PGE2 that are known to cause inflammation and pain [39]. Interestingly, these EVs were found to transport arachidonic acid to the FLS for conversion into inflammatory mediators by FLS-derived COX-2 [38]. In addition to the enzymes that are induced, the proinflammatory nuclear transcription factors NF-κB, AP-1, and JNK are also increased in the FLS after treatment with EVs isolated from TNF-α-treated T cells and monocytes [38]. Inhibition of the NF-κB and AP-1 pathway prevents the activation of mPGES-1 but not COX-2 [38]. However, inhibition of JNK did block the activation of COX-2, indicating that the JNK pathway is responsible for microsomal activation of COX-2 and subsequently PGE2 [38].

EVs derived from TNF-α-treated monocytes and T cells can directly stimulate the FLS secretion of inflammatory mediators such as IL-6 and IL-8 [38]. These mediators are known to contribute substantially to inflammation in patients with RA, and blocking IL-6 is one treatment for RA resistant to conventional therapies [40]. An additional finding that suggests EVs play a significant role in the inflammation caused by RA is that platelet-derived EVs were found in the synovial fluid of patients with RA, and not found in patients with OA [41]. The glycoprotein VI receptor, which is a collagen receptor, is the key receptor for the induction of EV production by platelets [41]. The EVs from the platelets were shown to promote inflammation via the IL-1 receptor in FLS [41].

Toll-like receptor 4 (TLR-4) and its coreceptor MD-2 are known to contribute to inflammation in a large number of diseases, including RA [42–46]. Further supporting the role of this receptor in the development of RA are studies showing that mice deficient in TLR4 are protected from developing experimentally induced arthritis, and that blocking the TLR4 receptor is a successful therapeutic in treating experimentally induced arthritis [43–46]. LPS is known to activate the signal transduction of TLR4, but the search for endogenous activators of this pathway has previously been unsuccessful. Plasma-derived EVs from patients with RA stimulated this receptor via a similar mechanism to LPS, that is by increasing activity of the TLR4 pathway significantly more than EVs from healthy subjects [42]. Further studies were carried out to confirm that it was truly through the TLR4 pathway, in which applying the same EVs to monocytes with a point mutation in the LPS binding domain of the receptor failed to induce inflammation [42]. Investigation into the specific mechanism of activation revealed that oxidized phospholipids in the membrane of EVs were responsible for the stimulation of the receptor [42]. Interestingly, when the effect of oxidized EVs on monocyte gene expression was examined, it was shown that the gene expression profile was markedly different than that of the cells stimulated with LPS [42]. The gene expression profile was analyzed in relation to genes associated with RA and found that there was substantial induction of inflammation resolving genes, notably IL-4 which promotes repair and decreases inflammation [42]. This differs from other work described previously which suggests the involvement of EVs in furthering inflammation. The EVs in this study seem to function as an oxidative-stress warning signal to the tissues to resolve inflammation. Of note, previous work has indicated that citrullinated immune complexes, a finding present in nearly 100% of RA patients, stimulate the TLR4 receptor to induce TNF-α significantly more effectively than uncomplexed citrullinated proteins [34]. The disruption of the balance of anti-inflammatory EV stimuli and inflammatory EV stimuli at the TLR- receptor represents a potential source of some of the inflammation associated with RA that deserves further investigation.

Another study has corroborated what Mancek-Keber et al. [46] reported about EVs helping to resolve inflammation. EVs were isolated from synovial fluid of patients with RA, and the proportions of EVs originating from neutrophils, monocytes, and T cells were characterized using different cell surface markers specific to each cell line [47]. Neutrophils contributed to the EVs in the synovial fluid but the concentration of all of the cell line-derived EVs was elevated significantly compared with the plasma [47]. A higher percentage of synovial fluid EVs contained the anti-inflammatory protein annexin A1 (ANXA1) compared with plasma-derived EVs [47]. ANXA1 has been shown to have anti-inflammatory effects, although the mechanism of action of this protein was not known previously [47–50]. A mouse model deficient in neutrophil EVs demonstrated twice the amount of cartilage loss when subjected to inflammatory arthritis compared with mice that had this mechanism intact [47]. ANXA1-containing EVs applied to chondrocytes in vitro activated anabolic genes, resulting in accumulation of ECM and a reduction in inflammatory cytokines IL-8 and PGE-2 [47]. This finding was supported by an in-vivo mouse study where ANXA1-containing EVs injected into the joint space of mice with experimental induced inflammatory arthritis resulted in significantly less cartilage destruction than the control group [47]. Mice given neutrophils via adoptive transfer demonstrated abundant EVs in the joint space but no neutrophils, indicating that the neutrophils deliver their EVs to the joint space without penetrating the synovial membrane [47]. Because little was known about the mechanism of action of ANXA1, further studies were undertaken to elucidate the mechanism. These studies demonstrated that ANXA1 is a ligand for the formyl peptide receptor 2 (FPR2/ALX) on chondrocytes, which when stimulated results in increase TGF-β by the chondrocytes [47]. The upregulation of ECM production is blocked by a specific FPR2 inhibitor, suggesting that the upregulation of ECM is truly caused by ANXA1 [47]. This upregulation occurred both with and without costimulation with IL-1β, indicating that the process is not suppressed by inflammation and making it an interesting therapeutic target for both RA and OA [47].

### Destruction of ECM

EVs derived from monocytes and T cells treated with TNF-α induce the production of large quantities of matrix metalloproteinase-1 (MMP-1), MMP-3, MMP-9, and MMP-13 by FLS [51–53]. MMPs, especially MMP-13, break down proteoglycans, such as aggrecan and collagen, in the ECM and are thought to be a major mechanism of cartilage destruction in RA [51, 53]. Interestingly, blocking the TNF-α and IL-1β receptor did not mitigate the response by FLS, indicating that EV-induced inflammation is independent of TNF-α-induced inflammation and ECM breakdown [51]. RA-derived FLS secrete EVs that contained high levels of ADAMTS-5 [54]. This further indicates that EVs released from synovial tissues have the capability to directly break down joint tissue, thereby further contributing to joint destruction. Moreover, EVs isolated from endothelial cells carry MMP-2, MMP-9, and MMP-14, indicating involvement of EVs in the breakdown of capillary membrane contributing to fluid build-up, swelling, and transfer of cells and proteins from the joint space into systemic circulation [55].

Hexosaminidase D and B-glucuronidase are enzymes with similar activity to aggrecanase, and are present in EVs in the joint space of patients with both RA and OA [56, 57]. While hexosaminidase enzymes generally have a wide substrate profile, making it hard to identify which particular enzyme causes the destruction, hexosaminidase D is elevated in synovial fluid EVs of both patients with RA and OA [56, 57]. B-glucuronidase has enzymatic activity in EVs derived from both RA and OA patients [56, 57]. Previously, this was thought to be a housekeeping gene but its localization in the EVs indicates that it is involved in regulation of ECM turnover [56, 57]. This represents yet another catabolic process in the development of RA that may involve EVs.

### miRNA delivery

**Fig. 1:**
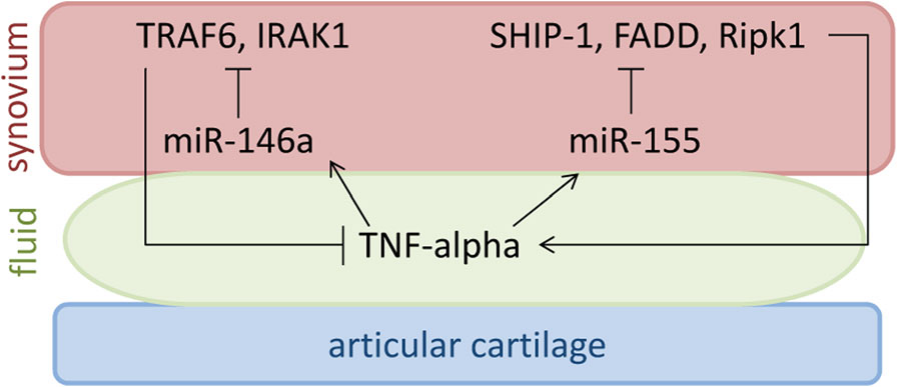
TNF-α in the joint fluid stimulates FLS to increase microRNAs miR-155 and miR-146. miR-155 stimulates production of Src homology 2-containing inositol phosphatase-1 (*SHIP-1*), Fas-associated death domain protein (*FADD*), and Serine-threonine kinase 1 (*Ripk1*) to promote inflammation and increase TNF-α production by the FLS. miR-146a downregulates TNF receptor associated factor 6 (*TRAF6*) and IL-1 receptor associated kinase 1 (*IRAK1*) to suppress inflammation and decrease TNF-α production. Additionally, these miRNA are found in the joint space of patients with RA and increased in EVs released by dendritic cells in response to inflammation

It is now recognized that miRNAs play a role in RA pathophysiology. The most well-known miRNAs involved in the pathophysiology of RA are miR-155 and miR-146a (Fig. 1). miR-155 is upregulated in the FLS of patients with RA compared with OA patients and normal controls, and inhibition of miR-155 in FLS results in decreased TNF-α production in vivo [58, 59]. Additionally, miR-155 knockout mice are resistant to the development of collagen-induced arthritis, while overexpression of miR-155 in mice results in a chronic inflammatory state with increased production of inflammatory cytokines [58]. miR-155 is stimulated by TNF-α and LPS, further implicating its role in the development of RA [60]. Modulation of inflammation is accomplished by targeting the transcripts of Src homology 2-containing inositol phosphatase-1 (SHIP-1), Fas-associated death domain protein (FADD), IκB kinase ε (IKKε), and serine-threonine kinase 1 (Ripk1), which interact with the TNF-α receptor and upregulate TNF-α translation [58, 60]. Interestingly, overexpression of miR-155 results in inhibition of MMP-13 production in response to inflammatory stimuli [59]. miR-146a is upregulated in the FLS of patients with RA compared with patients with OA and normal controls [61]. In-vivo studies show that miR-146a is upregulated in FLS by TNF-α and by IL-1β [61]. miR-146a overexpression suppresses IL-6 and IL-8 secretion and downregulates the IL-1 receptor associated kinase 1 (IRAK1) and TNF receptor associated factor 6 (TRAF6) genes [61–63]. Overexpression mouse models exhibit a decreased immune response by monocytes when challenged with LPS, while knockout mice models show an increased production of TNF-α, Il-6, and IL-1β when treated with LPS [64].

miR-155 and miR-146a are also found in EVs released by dendritic cells and taken up by all immune cells [65]. miR-146a reduces inflammatory gene expression in dendritic cells while miR-155 promotes inflammatory gene expression [65]. This finding was replicated in a mouse model, supporting the functional importance of this pathway in mediating inflammation [65]. miR-155 knockout mice were given an LPS challenge following administration of EVs loaded with miR-155. The control mice exhibited no inflammation following the challenge whereas the EV-treated mice exhibited inflammation and had detectable levels of miR-155 in all immune cells in addition to elevated TNF-α and IL-6 levels [65]. miR-146a knockout mice that underwent the same experiment showed large levels of inflammation and the EV-loaded miR-146a-treated mice showed lower levels of TNF-α and IL-6 [65]. Again, all immune cells had detectable levels of miR-146a, demonstrating the importance of this pathway in modulating the immune response [65]. Furthermore, the presence of EVs containing miR-155 and miR-146a in an inflammatory state suggests possible crosstalk between dendritic cells and FLS. While the exact mechanism by which EVs are taken up by immune cells may be cell specific, a few different mechanisms have been demonstrated. It is clear that EVs do possess different adhesion molecules that facilitate their interaction with immune cells such as the integrins ανβ3 and ανβ5, ICAM1, and LFA1 [66–71]. Interestingly, it has been shown that MHC II and ICAM1 are required for EVs to activate naïve T cells [70]. Membrane fusion at the cell surface has also been demonstrated as one uptake mechanism of EVs, which results in the direct transfer of proteins to the plasma membrane surface. Current literature indicates that a majority of the EVs are phagocytosed [11, 72–74]. Alternatively, macropinocytosis has also been shown to be a potential mechanism of uptake [11, 75]. Once inside the cell, the EV either fuses membranes with the endosome or is degraded in the lysosome [11]. If fusion occurs, the proteins can be recycled and presented on the accepting cells membrane [11]. Further work is needed to determine the exact mechanism of EV uptake by specific cell types.

### Biomarker of disease

Current data suggest that EVs may serve as biomarkers for rheumatic diseases. Serum levels of EVs are elevated in RA compared with healthy controls [76]. Additionally, the protein content of EVs from patients with RA is altered [27]. Synovial fluid and serum EVs from patients with RA contain 10 different proteins specific to RA EVs that are not found in patients with OA or reactive arthritis [27]. Over half of these proteins were also found in the citrullinated form, further increasing the specificity of these proteins as diagnostic tools [27]. Recent recognition of the differences in miRNA EV signatures in different disease states provides a promising new method to detect RA earlier and more accurately [21–23, 77]. An extensive profiling of EVs from the plasma and joint fluid of RA patients and healthy controls might reveal new biomarkers associated with RA disease progression and response to treatment.

### Extracellular vesicles as therapeutic vehicles for the treatment of RA

EVs have already shown therapeutic potential in patients with RA. In a collagen-induced arthritis model, EVs containing IL-10 and EVs derived from dendritic cells treated with IL-10 have strong anti-inflammatory properties when isolated, purified, and injected periarticularly [78]. Surprisingly, this change occurs not only at the injected joint but also at the contralateral joint even when injected locally, indicating that there is systemic circulation of EVs [78]. This finding was replicated when the EVs were injected systemically [78]. Local injection in the joints of the murine model with dendritic cells transfected with IL-10 suppresses inflammation both locally and in contralateral joints [78].

Vanniasinghe et al. [79] recently described targeting liposomes, small artificial vesicles with similar properties to EVs, to FLS. Therapeutically, liposomes are similar to EVs in the sense that they can be loaded with cargo, are biocompatible, and demonstrate the ability to get into cells [80]. Liposomes differ from exosomes in that their membranes are significantly less complex, their circulation time is adjustable depending on the composition, and the ability to target cells may be limited [80]. Vanniasinghe et al. [79] successfully delivered immunosuppressive therapy in the form of glucocorticoids to the synovial membrane and saw a dramatic reduction in inflammation in a collagen-induced arthritis model. This demonstrates a novel therapeutic technique that can be used to target inflamed synovial joints without the unwanted side-effect profile of steroid medication. Systemic side effects have been a huge hurdle in therapeutics for RA, particularly the newer biologics. While these therapies are useful for controlling the disease symptoms of RA, they leave patients chronically immunosuppressed, which can increase risk for infection. Targeting synovial joints systemically by liposomes for delivery of therapeutics such as biologics could result in improved efficacy of current treatment, drastically improved side effect profile of current treatments, and an opportunity for new therapeutics that can further alter the course of RA.

The recent studies suggesting that EVs serve as a warning signal and are actively involved in reducing inflammation obviously differ from those studies showing that EVs contribute to the inflammation of RA. Taken together it is apparent that the role of EVs in the pathogenesis of RA is dependent on the type of cells from which the EVs are derived. The studies indicating that EVs are involved in a physiologic response to limit inflammation further emphasize the potential utility of EVs as therapeutics for RA. Developing a therapeutic that can mimic or amplify a natural response to decrease inflammation represents a promising therapeutic target.

### Extracellular vesicles in the development and pathogenesis of OA

A role for EVs in OA is less well documented than for RA. The pathogenesis of OA is complex, and both the chondrocytes themselves and the extracellular matrix (ECM) are crucial to maintain healthy articular cartilage [81, 82]. The ECM is vital to the maintenance of articular cartilage because it has a very low cell density, which is critical for the functional properties of the tissue [82]. The ECM is largely made up of type II collagen and proteoglycans, in particular aggrecan [83]. Chondrocytes are solely responsible for the synthesis of aggrecan, which subsequently becomes articular cartilage [82]. FLS are responsible for secreting joint fluid that lubricates the articular cartilage. In healthy articular cartilage, a balance between synthesis and breakdown of the ECM maintains cartilage integrity [82, 84]. This specific balance between synthesis and degradation of the ECM is disturbed in the pathological condition of OA [84], such that ECM synthesis can no longer compensate for the loss of matrix structural integrity. The disease process progresses to the point where clinical symptoms arise, such as pain, bone-on-bone grinding, osteophyte formation, and joint space narrowing [85]. The specific mechanism by which that balance is disturbed is multifactorial and has not yet been fully elucidated. MMPs are a family of proteinases that are believed to contribute largely to the breakdown of ECM, in particular MMP-13 [86–88]. Currently MMP-13 is thought to be the major mediator of ECM breakdown that causes the majority of the pathology seen in OA and is produced by both chondrocytes and FLS [89–91]. MMP-13-deficient mice are resistant to collagen and aggrecan breakdown, which subsequently prevents cartilage erosion [91]. This enzyme is induced by the inflammatory cytokines IL-1β and TNF-α in the joint space [92, 93].

### Role of EVs in communication between FLS and chondrocytes

**Fig. 2:**
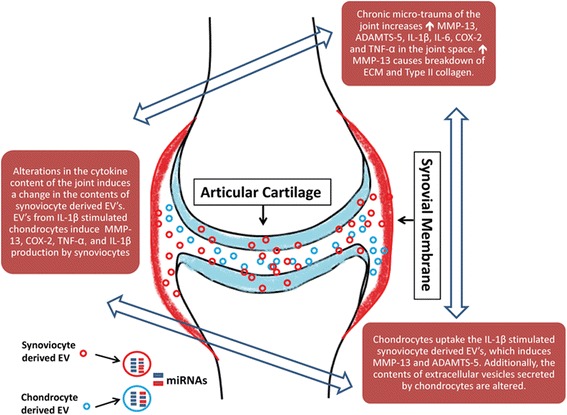
Proposed mechanism of EV communication between FLS and chondrocytes in OA. EVs from FLS stimulated with inflammatory cytokines in the synovial fluid are released into the synovial fluid act on chondrocytes to increase MMP-13 and ADAMTS-5. EVs from chondrocytes stimulated with inflammatory cytokines are released into the joint space and increase MMP-13, COX-2, IL-1β, and TNF-α. This positive feedback cycle leads to further breakdown of the articular cartilage ECM. *COX-2* cyclooxygenase 2, *ECM* extracellular matrix, *EV* extracellular vesicle, *miRNA* microRNA, *MMP* matrix metalloproteinase

OA involves many different cell types, and until recently little has been known about cellular communication between different cell lineages. EVs serve as a communication pathway between different tissue types and between different cell types, and thus represent a crucial step in the regulation of the disease process (Table 2) [17, 18]. When EVs derived from chondrocytes treated with IL-1β are applied to FLS, there is a nearly 3-fold increase in MMP-13 production as compared with EVs derived from chondrocytes without IL-1β stimulation (Fig. 2) [94]. Additionally, there is markedly increased production of IL-1β, TNF-α, and COX-2 by the synovial membrane, indicating that the EVs are playing a role in the inflammatory component of OA [94].

**Table 2:** Proposed roles of extracellular vesicles in osteoarthritis

Process	Description
Communication between FLS and chondrocytes	FLS EVs are known to be secreted into the joint space and are taken up by chondrocytes. EVs isolated from chondrocytes treated with inflammatory cytokines are known to increase inflammatory cytokine production and MMP-13 production by FLS. EVs isolated from FLS treated with inflammatory cytokines are known to increase inflammatory cytokine production and MMP-13 production by chondrocytes [25, 94, 98]
Biomarker	Differences in content of synovial fluid and plasma EVs can serve as a biomarker for disease. miR-200c is elevated compared with non-OA patients [98]
Therapeutic	Deliver miRNA to cells altering response to inflammation. Potential to target the reduction of MMP-13 production using miRNA. Additionally, EVs could be used to induce chondrogenesis.

*EV* extracellular vesicle, *FLS* fibroblast-like synoviocytes, *miRNA* microRNA, *MMP* matrix metalloproteinase, *OA* osteoarthritis

OA chondrocytes treated with EVs derived from FLS exposed to IL-1β upregulate MMP-13 and ADAMTS-5 and downregulate type II collagen [25]. These findings suggest that there is a positive feedback loop in the joint space, between the FLS and the chondrocytes, that promotes inflammation (Fig. 2). Additionally, EVs from IL-1β-stimulated fibroblasts have an increased concentration of IL-6, MMP-3, and VEGF [25]. When these EVs were applied to mouse femoral cartilage explants, the IL-1β-treated FLS EVs induced greater proteoglycan production than the EVs from IL-1β-naïve FLS [25]. However, both sets of EVs stimulated proteoglycan production more than media without EVs [25]. The IL-1β-treated FLS EVs also induced angiogenesis significantly more than the EVs from FLS with no treatment [25].

### MicroRNA profiling of EVs in OA

EVs from IL-1β-treated FLS were also profiled for differences in miRNA expression profiles. A total of 340 miRNAs were found to be upregulated in cells treated with IL-1β while only 11 miRNAs were found to be upregulated in the EVs, revealing selective packaging of miRNAs into EVs by the FLS [25]. Thirty-nine miRNAs were found to be downregulated in the EVs while only 24 were downregulated in the cell [25]. Of the 11 miRNAs upregulated in the EVs, only five of them were also upregulated in the cell [25]: miR-500B, miR-4454, miR-720, miR-199b, and miR-3154. Among these, miR-4454, miR-720, and miR-199b are the most well studied. miR-199b is increased during chondrogenesis and is decreased during senescence of mesenchymal stem cells [95]. miR-4454 is increased with TNF-α stimulation and is a target of NF-κB [96]. miR-720 promotes cell migration but its study in relation to the musculoskeletal system is limited [97].

**Fig. 3:**
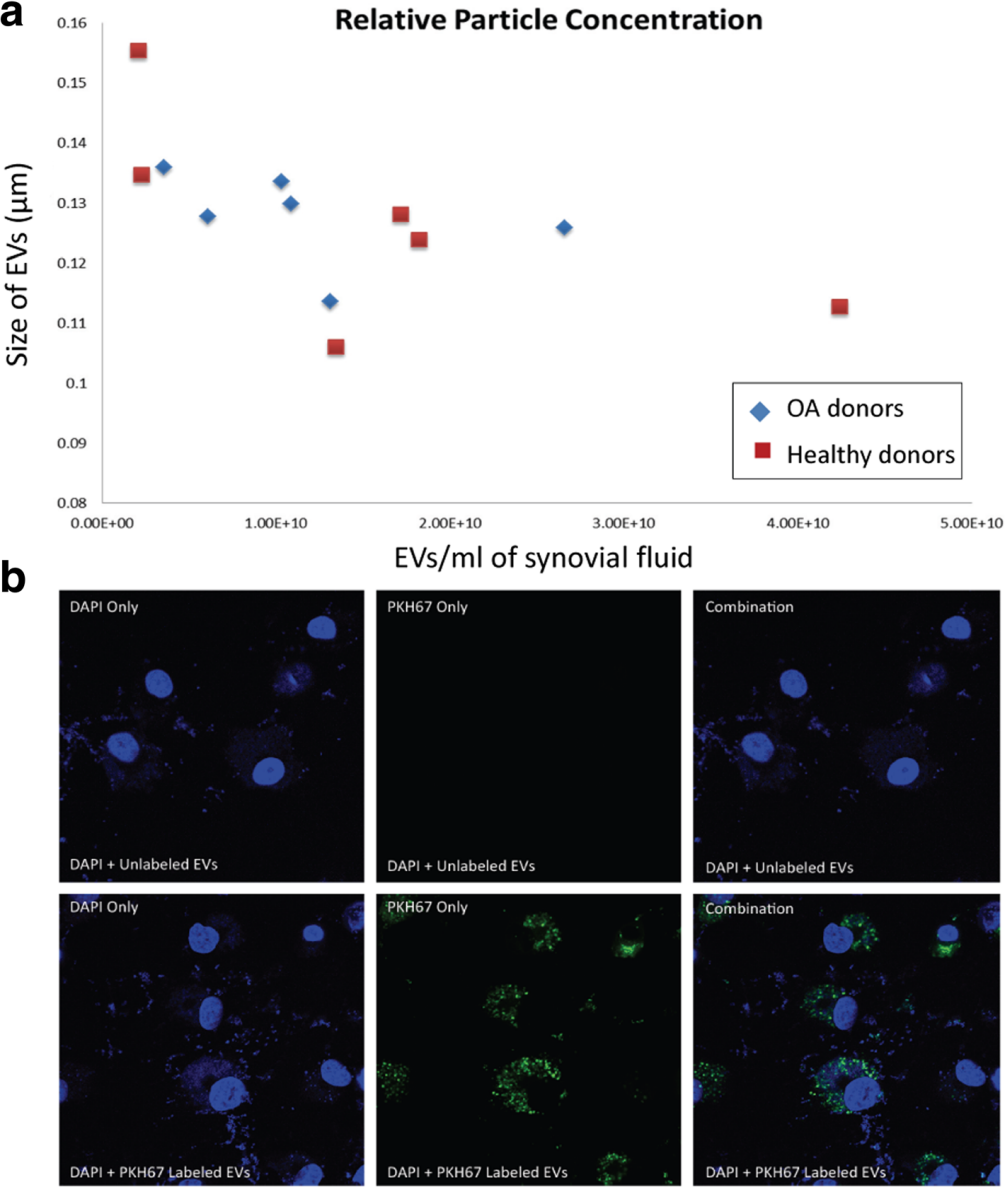
**a** Concentration of EVs in synovial fluid (*x* axis) versus the average size of EVs (*y* axis). There was no significant difference in either measurement between EVs from OA patients and EVs from normal patients. **b** *Top row*, chondrocytes treated with DAPI and unlabeled EVs; *bottom row*, chondrocytes treated with DAPI and PKH67-labeled EVs. *Left column*, only DAPI labeling; *middle column*, only PKH67 labeling; *right column*, combination of DAPI and PKH67 labeling. *EV* extracellular vesicle, *OA* osteoarthritis

We recently examined EVs from the synovial fluid of patients with OA and without OA [98]. Neither the concentration (OA: 1.18 × 10^10^ particles/ml, *n* = 6; non-OA: 1.59 × 10^10^ particles/ml, *n* = 6) nor the size (OA: 0.128 μm, *n* = 6; non-OA: 0.127 μm, *n* = 6) of nanoparticles differed between the groups (Fig. 3a) [98]. Chondrocytes treated with labeled EVs isolated from the synovial fluid of OA patients indicate that synovial fluid-derived EVs are readily endocytosed by chondrocytes (Fig. 3b) [98]. This further suggests that EVs carrying miRNAs and other cargo impacting chondrocyte cell death or ECM degradation may contribute to the pathogenesis of OA. Profiling of EV cargo by PCR array showed that miR-200C was increased 2.5-fold in EVs from OA patients [98]. This miRNA is known to be upregulated with oxidative stress and targets the zinc finger binding transcription factor ZEB1, resulting in repression of its transcription [99]. ZEB1, also known as delta EF1, plays a prominent role in maintaining articular cartilage in adults and is expressed at high levels in articular cartilage [100]. Mice without Zeb1 have severe skeletal deformities, because this transcription factor is known to participate in bone formation [101]. Interestingly, ZEB1 represses the type II collagen promoter and decreases the levels of type II collagen transcription [102]. miR-200c expression is suppressed by IL-6 and plays a role in mitigating IL-6 mediated inflammation [103]. Transfer of miR-200c represents one way in which FLS communicate with chondrocytes to maintain articular cartilage and is a potential targetable mechanism to reduce inflammation and increase chondrocyte synthesis of type II collagen. Future studies will be directed at evaluating EV-derived miR-200c as a potential biomarker for tracking the development and progression of OA (Table 2).

### Extracellular vesicles as therapeutic vehicles for the treatment of OA

miRNA regulation of chondrocyte-specific genes represents a potential therapeutic target for OA. Currently, OA is generally managed with NSAIDs, behavioral modifications, and eventual replacement of the joint with prosthesis. Existing therapeutic approaches are not effective for altering progression of the disease, unlike the disease-modifying agents used to treat RA. miRNA regulation represents a novel therapeutic approach that has the potential to halt the progression of the disease by blocking the induction and actions of MMP-13 and promoting chondrocyte health. Synovial joints present a unique environment to deliver small molecule therapeutics because they are largely isolated from the rest of the body. This represents another advantage of using these molecules to treat OA because the synovial fluid is a relatively insulated space, which would limit the molecules from being delivered systemically if the dosage was adequately controlled. Additionally, with the recent identification of a method to target FLS, there is already a method to target synovial joints for therapeutics [79]. The identification of a method to specifically target chondrocytes would open up the possibilities of therapeutics even further. miRNA is normally degraded in the joint space, but EV delivery can improve the short-term stability of miRNA by protecting these small mRNAs from breakdown. Previous studies have demonstrated that different miRNAs can be loaded into human-derived EVs [104]. Suspension of therapeutically altered EVs in a hydrogel-scaffold or chondroitin sulfate sponge could result in a stable long-term delivery system of miRNA to an isolated synovial joint. Further studies need to be carried out to better define the safest and most effective way to target this process.

## Conclusions

Establishing biomarkers that can identify the development of joint disease at the earliest stages will benefit patients that may ultimately go on to develop RA and OA. Most of the joint destruction in RA occurs early in the disease and for this reason treatment is not delayed until the onset of symptoms [105]. This underscores the need for further research into EV profiling for RA patients. Because there is currently no cure for RA, identifying the disease earlier and enrolling the patients in treatment before the symptoms become severe is the most useful way to prevent morbidity and mortality in patients with RA. Additionally, the recent research involving EV mediation of the immune system and inflammatory response further indicates the need for more investigation into the role of EVs in RA. Future work into the mechanism of EV-mediated immune response modulation with regard to RA has the potential to not only reveal meaningful discoveries into the pathogenesis of disease, but also new ways to therapeutically target the disease. Recent work into EVs has already revealed a mechanism by which to target synovial membranes using EVs [79]. Additional investigation needs to be done regarding the utility of this delivery method with the drugs currently available to treat RA.

The role of EVs in OA has provided a foundation to potentially create novel nonsurgical, disease-modifying treatments for OA. There are currently no therapeutic interventions that can reverse the process of OA. Using EVs to deliver specific miRNA known to reduce MMP-13 production in the joint space could decrease the amount of cartilage destruction, potentially tipping the balance in favor of cartilage synthesis. While there is a large amount of research regarding miRNA regulation of MMP-13, more work needs to be done with regard to the utility of EVs in reducing the destruction of ECM by MMP-13. Additionally, EVs with miRNAs that are known to promote chondrogenesis could help further increase the concentration of chondrocytes and replace the damaged chondrocytes. More research into the miRNA regulation of chondrogenesis could identify a potential miRNA formulation that increases chondrogenesis.

Crosstalk between the immune system and synovium in RA, and crosstalk between the synovium and articular cartilage in OA, are two important communication pathways that need further investigation to more fully understand the pathophysiology of RA and OA. EVs appear to be key messengers in these communication pathways, and future studies of EVs associated with joint disease may uncover new therapeutic opportunities and treatment strategies. A better understanding of the mechanism of EVs and the contribution of EVs to normal physiology and pathology will require an improved classification system for EVs and further standardization of the techniques used to isolate EVs. Key steps toward improving this classification have been made, such as the International Society for Extracellular Vesicles minimum requirements for definition of EVs, EV protein composition databases, and improvement in isolation techniques. However, continued commitment to this endeavor and collaboration between the scientists in the field is required to further our understanding of this critical communication mechanism and potential therapeutic revolution.

## Abbreviations

AIM: Apoptosis inhibitor of the macrophage
ANXA1: Annexin A1
COX-2: Cyclooxygenase 2
ECM: Extracellular matrix
EVs: Extracellular vesicles
FADD: Fas-associated death domain protein
FLS: Fibroblast-like synoviocytes
FPR2: Formyl peptide receptor 2
IKKε: IκB kinase ε
IRAK1: IL-1 receptor associated kinase 1
miRNA: MicroRNA
MMP: Matrix metalloproteinase-1
mPGES-1: Microsomal prostaglandin E synthase 1
OA: Osteoarthritis
PADs: Peptidylarginine deiminase family of enzymes
PGE2: Prostaglandin E2
RA: Rheumatoid arthritis
Ripk1: Serine-threonine kinase 1
SHIP-1: Src homology 2-containing inositol phosphatase-1
TRAF6: TNF receptor associated factor 6
